# Unilateral Partial Absence of the Fallopian Tube in a Female Patient With Infertility: A Case Report and Literature Review

**DOI:** 10.7759/cureus.33057

**Published:** 2022-12-28

**Authors:** Hiroaki Soyama, Satomi Okuguchi, Takayoshi Yoshida, Fumiaki Taniguchi

**Affiliations:** 1 Obstetrics and Gynecology, Takanohara Central Hospital, Nara, JPN

**Keywords:** salpingectomy, laparoscopic surgery, tubal infertility, hydrosalpinx, fallopian tube

## Abstract

Unilateral partial absence of the fallopian tube is rare, and its clinical importance in fertility is unclear. A 35-year-old nulligravid female patient with infertility was suspected to have a left hydrosalpinx on hysterosalpingography and sonography. Therefore, the patient underwent diagnostic laparoscopy. The left fallopian tube lacked the ampullary portion, and its proximal end had a hydrosalpinx. A left salpingectomy was performed, and the pathological finding was a unilateral partial absence of the ampullary portion of the fallopian tube with hydrosalpinx. Postoperatively, she conceived via in vitro fertilization-embryo transfer-and delivered a healthy baby. Hydrosalpinx is a well-known cause of infertility and can develop due to the partial absence of a fallopian tube. Furthermore, salpingectomy may be effective in improving fertility in female patients with a unilateral partial absence of the fallopian tube.

## Introduction

Partial tubal absence is a rare anomaly with unknown pathogenesis [1]. There are basically two etiologies: congenital and acquired [1]. The incidence of congenital unilateral partial absence of the fallopian tube has been suggested to be approximately one in 11,240 [2]. On the other hand, secondary pathogenesis has been reported, such as asymptomatic torsion followed by autoamputation, although its incidence is unclear [1].

Because the unilateral partial absence of the fallopian tube has only been reported in a small number of studies, its etiology, histopathology, and clinical impact on fertility are still unclear. It is usually asymptomatic, and most cases are accidentally detected during fertility examinations [1]. Therefore, the treatment of unilateral partial absence of the fallopian tube has not been established.

Herein, we report a rare case of an infertile female patient with a unilateral partial absence of a fallopian tube detected during the operation, together with a review of the literature. Written consent for publication was obtained from the patient.

## Case presentation

A 29-year-old nulligravid Japanese woman complained of one-year primary infertility and visited her first infertility clinic. The patient had no remarkable medical, surgical, or family histories. At that time, the patient underwent infertility examinations as follows: Blood tests, including biochemical analysis, immune serum analysis, blood coagulation analysis, and hormonal analyses such as thyroid and gonadotropin, all came back normal. Also, the patient didn’t have anti-sperm antibodies, and the chlamydia antibody test was also negative. Seminal analyses such as semen volume, sperm count, sperm concentration, sperm motility, and sperm morphology of her husband were within normal limits. The patient had hysterosalpingography, but she couldn't recall any of the results. She did not conceive after six years of trying to conceive through timed intercourse followed by artificial insemination by her husband with ovulation induction. 

**Figure 1:**
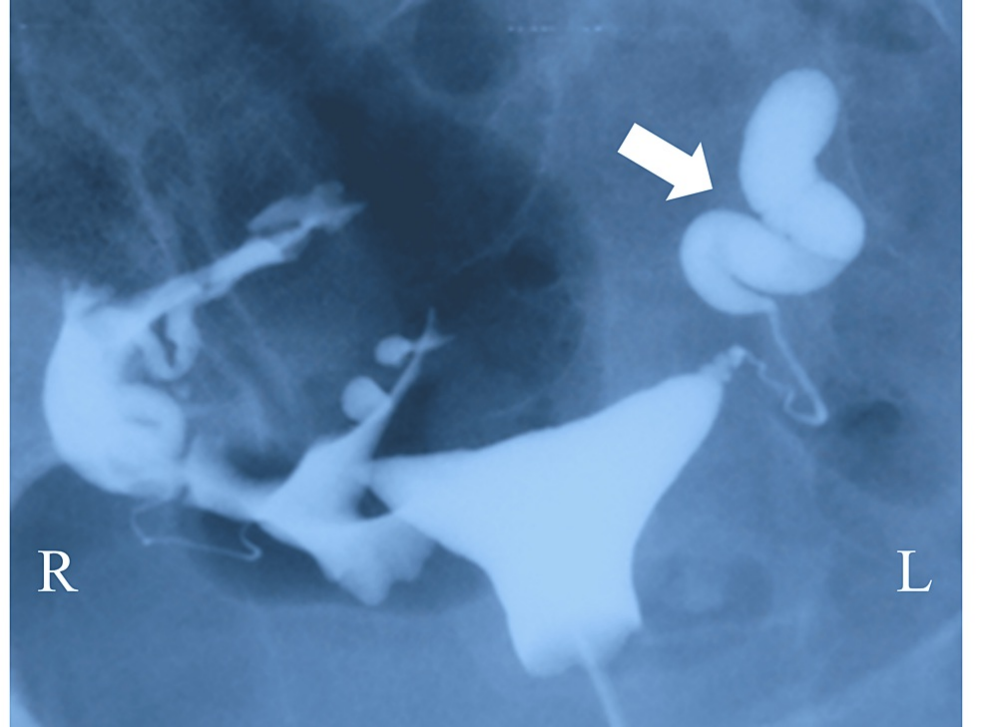
Hysterosalpingography before the operation. The left fallopian tube was abnormally swollen, and hydrosalpinx was suspected (indicated with an arrow).

Then, the patient visited another infertility clinic when she was 35 years old. The left hydrosalpinx was diagnosed using hysterosalpingography (Figure 1). Therefore, the patient was referred to our hospital for further treatment. The patient had no history of pelvic surgery, abdominal inflammation, or sexually transmitted diseases, and her menstrual cycle was regular. Her height, body weight, and body mass index (BMI) were 163 cm, 63.8 kg, and 25.6 kg/m2, respectively. There were no general findings such as pain, fatigue, or fever. Her abdomen was soft and flat. A pelvic examination revealed no abnormalities in her vulva, vagina, cervix, or uterus. Ultrasound examination revealed that her uterus was normal in size without any abnormalities, such as fibroids or congenital malformations such as the partial absence of the uterus. Hence, the patient was suspected to have tubal infertility due to hydrosalpinx. Even if she were to undergo in vitro fertilization (IVF) treatment, hydrosalpinx fluid would adversely affect IVF outcomes [3]. Hence, the patient underwent laparoscopic surgery for hydrosalpinx.

**Figure 2:**
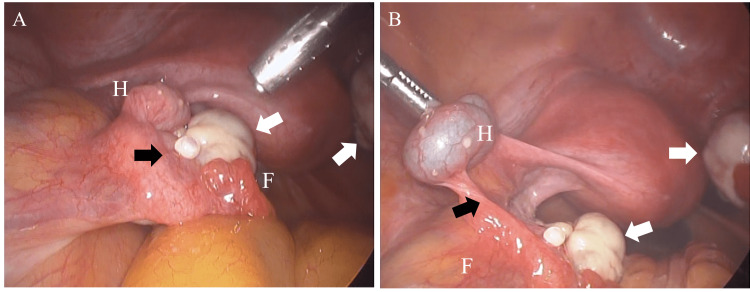
Images of diagnostic laparoscopy A) Before laparoscopic chromopertubation, the tube contained a hydrosalpinx tube. B) During laparoscopic chromopertubation, the indigo carmine dye did not pass through the left fallopian tube, and the dye was stuck at the proximal portion of the absence point. H: hydrosalpinx; F: fimbriae White arrows: normal ovaries; black arrows: completely absent ampullary portion

Although a normal uterus was identified during the operation, there was deep and superficial endometriosis found on the uterovesical pouch, Douglas pouch, and surface of the ovaries. The right fallopian tube and both ovaries appeared to be normal. However, the ampullary portion of the left fallopian tube was partially absent, and a hydrosalpinx was found on the proximal side of the left tube (Figure 2A). Although the indigo carmine dye passed through the right fallopian tube during laparoscopic chromopertubation, it was stuck at the left proximal portion of the absence point (Figure 2B). The patient was diagnosed with left hydrosalpinx because of the absence of the ampullary portion of the left fallopian tube, and a left salpingectomy was performed. Laser vaporization of endometriosis was also performed on the uterovesical pouch, Douglas pouch, and surface of the ovaries (revised American Society for Reproductive Medicine score [4], 20 points).

**Figure 3:**
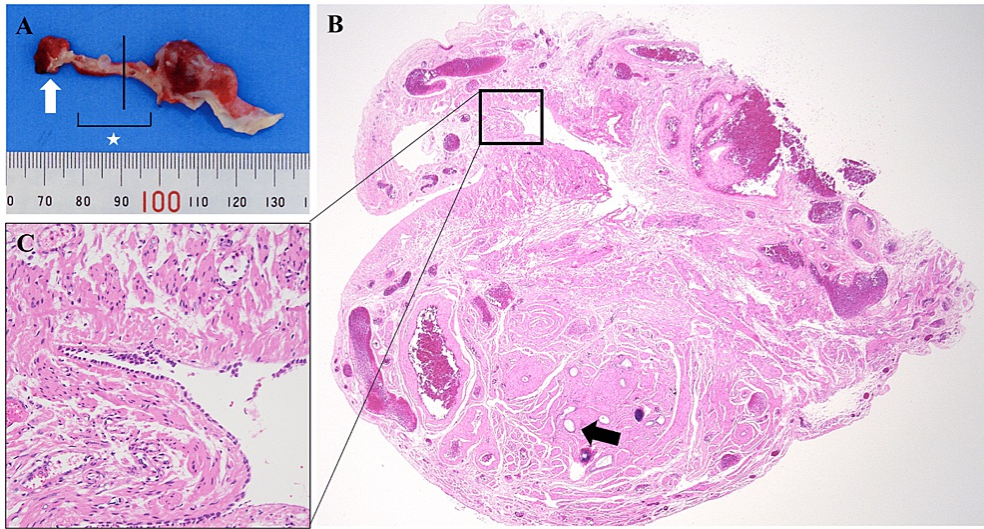
Pathological findings of the left tube A) macroscopic view of the tube. The tube was 60 mm in length and lacked the ampullary portion (white star). The white arrow is fimbriae. B) a microscopic view of A's black section. The absent portion of the fallopian tube contains a cleft structure (black window) and remnant Wolffian duct (black arrow) (H&E, ×20). C) a cleft structure was suspected as a tubal remnant (H&E, ×200).

Histopathological examination revealed that the left fallopian tube was 60 mm in length and lacked an ampullary portion (Figure 3A). Although the section of tubal absence contained Wolffian duct remnants, it lacked the normal fallopian tube structure (Figure 3B). Microscopically, cuboidal epithelial cells were found within the lining of the cleft structure, which was surrounded by double-layer smooth muscle cells (Figure 3C). Thus, although the cleft structure did not contain fimbria, this may suggest an atrophied tubal remnant. There was no endometriosis on the left fallopian tube, which resulted in a narrowed tube due to adhesion. The proximal side of the left tube was diagnosed as hydrosalpinx. The patient did not have any other congenital anomalies, such as those of the urogenital systems, including kidney agenesis, which was determined by ultrasonography and computed tomography scan. The patient didn’t undergo a karyotype examination.

After the surgery, the patient conceived via IVF frozen-thawed embryo transfer. At 41 weeks of gestation, the patient underwent a cesarean section due to labor arrest, and she delivered a male baby weighing 3376 g with Apgar scores of nine and 10 at one and five minutes, respectively.

## Discussion

Recently, three possible etiologies of partial tubal absence have been suggested [2]. The first is a defect in the development of the Müllerian and mesonephric systems that occurs in the local region of the genital ridge and caudal part [2]. The second is an asymptomatic torsion of the adnexa during the fetal period [2]. The third is tubal maldevelopment caused by ischemia due to a vascular accident [2]. In the present case, Müllerian and mesonephric system malformations were suspected. However, the patient had a normal ipsilateral ovary with no other systemic anomalies. Thus, the case was not congenital but rather a secondary condition that caused the tubal absence [5]. For the torsion hypothesis, absence often occurred in the proximal portion of the tube and affected the ipsilateral ovary [2,6]. Additionally, the patient had no history of acute abdominal pain. Considering these facts, torsion was not suspected in the present case. However, the absence of symptoms does not exclude the possibility of torsion that occurred during the fetal period or childhood [2], according to the histopathological findings. Therefore, in the present case, a partial vascular accident may have indirectly affected the insufficient canalization or caused partial atresia due to atrophy of the tube.

Although several studies have reported the absence of ovaries and/or fallopian tubes in a normal uterus, there have been few reports regarding the clinical significance of infertile patients with a unilateral partial absence of fallopian tubes with normal ovaries. Including the present case, we reviewed the literature pertaining to the clinical outcomes of patients with unilateral partial absence of the fallopian tube and normal ovaries who underwent laparoscopic surgery (Table 1) [5,7]. Of six patients, five (83%) were complicated with hydrosalpinx, and all the patients underwent laparoscopic tubal surgery. Four (67%) patients subsequently conceived, and three (50%) delivered healthy babies. According to this review, all patients with ampullary absence were treated with hydrosalpinx. Therefore, the absence of an ampullary segment may potentially lead to hydrosalpinx. Generally, infertility caused by hydrosalpinx requires surgical interventions, such as salpingostomy and salpingectomy, to improve fertility [8-10]. Hydrosalpinx fluid has adversely affected in vitro fertilization outcomes; therefore, it is important to eliminate its detrimental effect [3]. Although the efficacy of salpingostomy for infertile women with hydrosalpinx has been reported, the subsequent ectopic pregnancy rate was 10% [11]. In contrast, if a patient has infertility on the contralateral side of the fallopian tube, IVF treatment is required after salpingectomy [11]. Hence, salpingostomy or salpingectomy should be considered for infertile female patients with hydrosalpinx, depending on the patient’s background.

**Table 1:** The review of clinical outcomes of the patients with unilateral partial absence of fallopian tube with normal ovaries IVF: in vitro fertilization; NM: not mentioned

Reference	Case	Age (in years)	Infertility	Diagnosis	Hydrosalpinx	Treatment	Subsequent pregnancy	Pregnancy outcome
Johnston et al.,2003 [7]	1	31	Primary infertility for eight years	Absent left ampulla	Yes	Laparoscopic left salpingostomy	No	No
	2	31	Secondary infertility for 18 months (previous miscarriage)	Absent left ampulla	Yes	Laparoscopic left salpingostomy	Yes	Delivered a healthy baby
	3	35	Primary infertility for 25 months	Absent right ampulla	Yes	Laparoscopic right salpingostomy	Yes	First trimester
	4	28	Primary infertility for 18 months	Absent right ampulla	Yes	Laparoscopic right salpingostomy	Yes	Delivered a healthy baby
Uckuyu et al.2009 [5]		36	Primary infertility for 18 years	Absent Right distal tubal	No	Laparoscopic bilateral salpingectomy	Referred for IVF	NM
The present case		35	Primary infertility for seven years	Absent left ampulla	Yes	Laparoscopic left salpingectomy	Yes	Delivered a healthy baby

According to previous reports, a unilateral tubal anomaly may negatively affect the function of the other tube or the pelvic microenvironment [2,5,7]. Additionally, partial tubal absence has already lost its fertile function and has the potential to develop into hydrosalpinx. Therefore, although several studies have reported that the influence on ipsilateral ovarian function due to a decrease in blood flow after salpingectomy is still controversial [12,13], salpingectomy may become a feasible option for infertile women with a unilateral partial absence of the fallopian tube, regardless of hydrosalpinx.

In the present case, there was a small amount of endometriosis in the uterovesical and Douglas pouches. Generally, superficial peritoneal lesions, such as those of the fallopian tubes and ovaries, are more closely associated with infertility than endometrioma and deeply infiltrating endometriosis because of the occlusion of the tubal ostium, which compromises sperm passage [14]. Consequently, endometriosis, in this case, was considered to have had little effect on fertility.

## Conclusions

Unilateral partial absence of the fallopian tube is rare and may be associated with infertility, especially in patients with hydrosalpinx. Furthermore, unilateral partial absence of a fallopian tube may potentially develop into hydrosalpinx and may negatively affect the function of the other tube or the pelvic microenvironment; therefore, laparoscopic salpingectomy might be rational for infertile patients with unilateral partial absence of a fallopian tube, regardless of hydrosalpinx.

## References

[1] Yazawa H, Yabe M, Endo S, Hayashi S (2010). A case of congenital unilateral partial absence of fallopian tube. Fukushima J Med Sci.

[2] Pabuccu E, Kahraman K, Taskın S, Atabekoglu C (2011). Unilateral absence of fallopian tube and ovary in an infertile patient. Fertil Steril.

[3] Tsiami A, Chaimani A, Mavridis D, Siskou M, Assimakopoulos E, Sotiriadis A (2016). Surgical treatment for hydrosalpinx prior to in-vitro fertilization embryo transfer: a network meta-analysis. Ultrasound Obstet Gynecol.

[4] (1997). Revised American Society for Reproductive Medicine classification of endometriosis: 1996. Fertil Steril.

[5] Uckuyu A, Ozcimen EE, Sevinc Ciftci FC (2009). Unilateral congenital ovarian and partial tubal absence: report of four cases with review of the literature. Fertil Steril.

[6] Grover S (2007). Torsion causing interruption of the ampullary portion of the fallopian tube. Fertil Steril.

[7] Johnston AC, McComb PF (2003). Fertility potential of women with congenital ampullary atresia of the fallopian tube. Fertil Steril.

[8] Melo P, Georgiou EX, Johnson N (2020). Surgical treatment for tubal disease in women due to undergo in vitro fertilisation. Cochrane Database Syst Rev.

[9] Strandell A, Lindhard A (2002). Why does hydrosalpinx reduce fertility? The importance of hydrosalpinx fluid. Hum Reprod.

[10] Johnson N, van Voorst S, Sowter MC, Strandell A, Mol BW (2010). Surgical treatment for tubal disease in women due to undergo in vitro fertilisation. Cochrane Database Syst Rev.

[11] Chu J, Harb HM, Gallos ID, Dhillon R, Al-Rshoud FM, Robinson L, Coomarasamy A (2015). Salpingostomy in the treatment of hydrosalpinx: a systematic review and meta-analysis. Hum Reprod.

[12] Chan CC, Ng EH, Li CF, Ho PC (2003). Impaired ovarian blood flow and reduced antral follicle count following laparoscopic salpingectomy for ectopic pregnancy. Hum Reprod.

[13] Orvieto R, Saar-Ryss B, Morgante G, Gemer O, Anteby EY, Meltcer S (2011). Does salpingectomy affect the ipsilateral ovarian response to gonadotropin during in vitro fertilization-embryo transfer cycles?. Fertil Steril.

[14] Tanbo T, Fedorcsak P (2017). Endometriosis-associated infertility: aspects of pathophysiological mechanisms and treatment options. Acta Obstet Gynecol Scand.

